# Fano resonance between coherent acoustic phonon oscillations and electronic states near the bandgap of photoexcited GaAs

**DOI:** 10.1038/s41598-018-35866-7

**Published:** 2018-12-07

**Authors:** M. Vinod, G. Raghavan, V. Sivasubramanian

**Affiliations:** 1 Materials Science Group Indira Gandhi Centre for Atomic Research, Kalpakkam, 603 102 Tamil Nadu India; 2 Homi Bhabha National Institute, Mumbai, India

## Abstract

Impulsive photo-excitation of solids results in a travelling strain pulse which manifests itself as coherent acoustic phonon oscillations. These oscillations have been extensively studied using time-resolved pump-probe spectroscopy. In the present work, we report the generation of extremely long-lived, coherent longitudinal acoustic phonon oscillations in intrinsic GaAs (100), with clear and unambiguous evidence of Fano interference between these oscillations and the continuum of electronic states close to the bandgap. Fano resonance is a widespread phenomenon observed in atomic systems and condensed media that arises from quantum interference between a continuum of quantum states and a discrete quantum state. Among other techniques, Fano resonance has been investigated with respect to optical phonons studied with Raman Spectroscopy. In the present work, we investigate Fano resonance in coherent phonon oscillations generated without the aid of any capping layer, dopants or substrate/interface effects. Since Fano resonance is sensitive to changes in electronic structure, doping and defects, these observations are important to the field of picosecond ultrasonics which is used for non-destructive depth profiling of solids and for carrier diffusion studies.

## Introduction

In a now classical work, asymmetric non-Lorentzian lineshapes of optical spectra were originally investigated by Fano in the context of autoionization in He atoms^1^. Fano resonance have since been reported in the spectroscopy of a wide variety of condensed matter systems like metals, semiconductors, ferroelectrics and superconductors, quantum well structures including those of nanostructured systems^2–10^. It is fairly well-established that Fano interference occurs due to the excitation of a discrete state and that of a continuum of states that interacts with it^1^. In metals, Fano resonance occurs due to the coupling between the discrete optical phonon and the broad electronic continuum of intra-band electronic density of states. On the other hand, in semiconductors, the interference between the discrete inter-band transitions and the broad phonon continuum results in the asymmetric Fano line-shape of the optical phonons^5,6^. In complex oxide ferroelectrics, due to the interaction with the polarization continuum, Fano like features are observed in the optical phonon spectra^11^. In the case of semiconductors like Si, the presence of Fano-like interactions were first established in heavily doped p-type silicon from the fact that lineshapes of Raman and infrared spectra of optical phonons were asymmetric, whereas the lineshapes are completely symmetric (Lorentzian) for lightly doped specimens^5,6^, The inference drawn from these studies was further bolstered by time-domain investigations of optical phonons using pump-probe spectroscopy^7^. The existence of interactions between electronic states with coherent optical phonon states resulting in Fano lineshape was established in metals like bismuth^8^, zinc^9^ and Te^10^ with pump-probe measurements. Recently Fano resonance has been extensively studied in nanophotonics, plasmonics, photonic crystals and meta materials^12–14^. Fano enhanced optical transmission and suppression have been observed in periodic array of sub wavelength holes in SiC film^13^. Controlling the Fano resonance in these artificial structures finds applications in optical sensing, switching, lasing and slow wave devices^15–18^.

**Figure 1:**
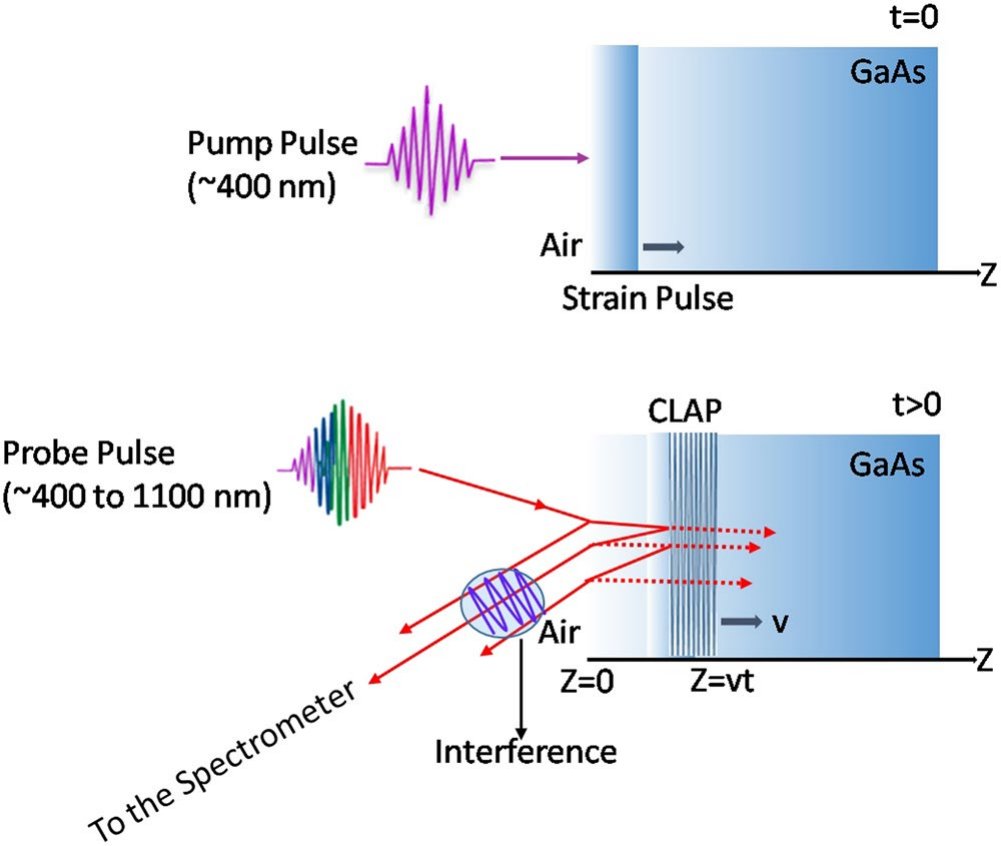
Schematic representation of the two-color pump-probe spectroscopy**:** creation of Coherent Longitudinal Acoustic Phonon (CLAP) up on the excitation with pump pulse and detection using the multi color probe beam. Reflections from both the surface and CLAP oscillation will interfere with each other and cause oscillations in the probe beam output.

Coherent acoustic phonon oscillations are generated under photoexcitation of the electronic ground state with femto-second laser pulses in many solid materials. Coherent acoustic phonon oscillations have been investigated in metals^19–22^, thin films & heterostructures^23–28^, oxides^29,30^, elemental^31^ and compound semiconductors^32–41^ using pump-probe spectroscopy. In transient pump-probe experiments, a high-energy pump pulse excites the electronic ground state of the specimen, creating a non-equilibrium concentration of hot photoexcited charge carriers. The relaxation of the hot photoexcited charge carriers occurs within a couple of picoseconds, giving rise to a transient peak in the differential reflectance of the probe pulse. This sharp transient is followed by an oscillatory tail in the probe reflectance as a function of delay time. These oscillations result from the travelling strain wave generated by internal stresses produced by the pump pulse. The refractive index becomes discontinuous at the travelling strain wave and as a result the probe pulse is reflected by it. This process is schematically illustrated in Fig. 1. The characteristics of CLAP wave generated in the sample is exquisitely sensitive to variations in the complex dielectric constant, making it an effective non-destructive depth profiling tool for investigating multilayers, defect concentrations and impurities^36,37^.

The mechanisms leading to CLAP generation are fairly well-understood through phenomenological models^38,39^. There are four possible sources of stress generation in solids resulting from optical pumping: 1. Thermoelastic (TE), 2. Deformation Potential (DP), 3. Piezoelectric and 4.that aided by built-in surface/interface induced stresses. While TE is the dominant process in metals, it is the DP process that governs the CLAP generation in semiconductors^39^. Since the DP process is less efficient than TE, CLAP waves are weaker in semiconductors. However the DP stress, when is enhanced by other coupling mechanism aids the generation of CLAP. For instance, the coupling between strain and the internal electric field as in the case of GaN/InGaN^40,41^ and GaAs (111), giving rise to inverse piezoelectric effects^42^ aids the generation of CLAP. But, for GaAs (100) there is little piezoelectric contribution making the direct generation of CLAP difficult. However, CLAP was successfully generated by capping GaAs with 20 nm GaSb layers^43^. The small band gap of GaSb (0.8 eV) efficiently absorbs the pump pulse thereby acting as a transducer. Transient CLAP oscillations have also been observed in GaAs/GaAlAs heterostructures grown by molecular beam epitaxy^28^. Wright *et al*.^34^ have reported observations of CLAP in a thin wafer of GaAs bonded to an acoustically mismatched substrate, which generates additional internal stresses.

In the experimental studies carried out so far, Fano resonance has been reported mostly on the spectra of optical phonons. Surprisingly there are no studies of an analogous interaction in the case of coherent acoustic phonon oscillations (CLAP). In this report, we present, what is to the best of our knowledge, the first observation of Fano resonance between the electronic states and CLAP oscillation in GaAs single crystal. We have used the two-colour pump probe spectroscopy with 400 nm pump pulse to observe extremely long-lived CLAP oscillations in GaAs (100) single crystal wafers. Fourier transform (FT) of the phonon oscillation data shows interesting Fano-like interference effects near the renormalized bandgap. The present work provides strong evidence of Fano resonance between the electronic states near the bandgap and CLAP in GaAs.

## Methods

The pump-probe differential reflectance measurements reported in this paper were carried out on intrinsic undoped GaAs (100) oriented wafer polished on both sides. The 400 nm pump was generated through a tuneable Optical parametric Amplifier (OPA, Orpheus + SHG, Light Conversion) pumped by a 1030 nm 9 W regenerative amplifier (Pharos, Light Conversion) with a nominal pulse width of ~230 fs and at the repetition rate of 125 KHz. A customized, commercial pump-probe spectrometer Harpia (Light Conversion) was used in the present study. About 15% of the output at 1030 nm was used to pump a sapphire crystal to produce the white light probe with a wide spectral distribution between ~400–1100 nm. A 400 nm (3.1 eV) pump of average continuous power of ~12 mW with equivalent pulse energy of 96 nJ was focused to a spot size of ~250 μm at near normal incidence on the sample. The probe was focused well within the pump, ensuring the homogeneity of excitation over the region of the specimen interrogated by the probe pulse. The optics was adjusted and calibrated for zero initial delay between the pump and the probe. A high resolution quadrupled motorized delay line was used for delaying the probe in steps of picoseconds or less. The reflected probe was at small angle away from the normal and due care was taken to have a stable probe beam and which was characterized using a motorized spectrometer equipped with an array detector (Andor, SR-193i). Spectral calibration was carried out with Holmium standard and chirp correction for the reflected probe was done with the software. A probe delay step of 0.5 ps was used to collect the transient data for the first 20 ps and thereafter the step size was adjusted to 2 ps for the rest of the data collected up to 2000 ps. The differential reflectance of the probe $\frac{R-{R}_{0}}{R}$, where *R* and *R*_0_ are the probe reflectances in the presence and absence of the pump, are obtained using a mechanical chopper operating at 130 Hz with care being taken to discard data collected when the pump is partially on.

## Results and Discussion

For intrinsic GaAs along (100) direction, the relative contributions coming from the DP and TE processes can be obtained from the following considerations: As the pump size is substantially larger (about ~250 μm) than the penetration depth, a one dimensional treatment is adequate to model the stress generation process. Under these conditions, the only relevant component of the stress tensor is *σ*_*zz*_(*z*, *t*) along the direction normal to the sample surface. The thermal and deformation potential stresses are of the same sign in GaAs^34^ and they add up with their ratio being:

1 $$\frac{{\sigma }_{\,zz}^{\,DP}}{{\sigma }_{\,zz}^{\,TE}}=\frac{C}{3\beta }(\frac{\,{\partial }{E}_{g}}{\,{\partial }p})\frac{1}{(E-{E}_{g})}$$

using the relevant quantities and their standard values for GaAs in Table 1, we see that this ratio is 6.43 for pump energy of ~3.1 eV. Therefore, the major contribution to the stress is of an electronic origin and comes from the deformation potential in GaAs. Since this source of stress is weak, CLAP is difficult to observe in degenerate pump-probe measurements with pump and probe close to the band-gap energy. This is because, the penetration depth of the pump is of the order of microns for these wavelengths and the pump energy is therefore distributed over a larger volume and strong localized stress pulse is not created. This is not the case when the penetration depth is small as in the case of pump close to the ultraviolet region. In the present work, two factors that have aided the generation of CLAP are that the pump wavelength was 400 nm and the penetration depth was only ~15 nm and (E_*pump*_ − E_g_) ≈ 1.7 eV. This excess energy causes the generation of very hot photo-excited charge carriers. Further, we have also used high pump fluence. The high internal stress created by the high pump fluence would have resulted in the long lived CLAP waves.

**Table 1:** Physical parameters of GaAs.

Parameter	GaAs
Longitudinal sound velocity, v_s_ (cm/s)	4.73 × 10^5^
Mass density, ρ (g/cm^3^)	5.317
Thermal expansion coefficient, β (K^−1^)	5.73 × 10^−6^
Deformation potential, ∂Eg/∂p (eV/Pa)	9 × 10^−11^
Band gap, E_g_ (eV)	1.424
Specific heat per unit volume C_v_ (J cm^−3^/°C)	0.33

**Figure 2:**
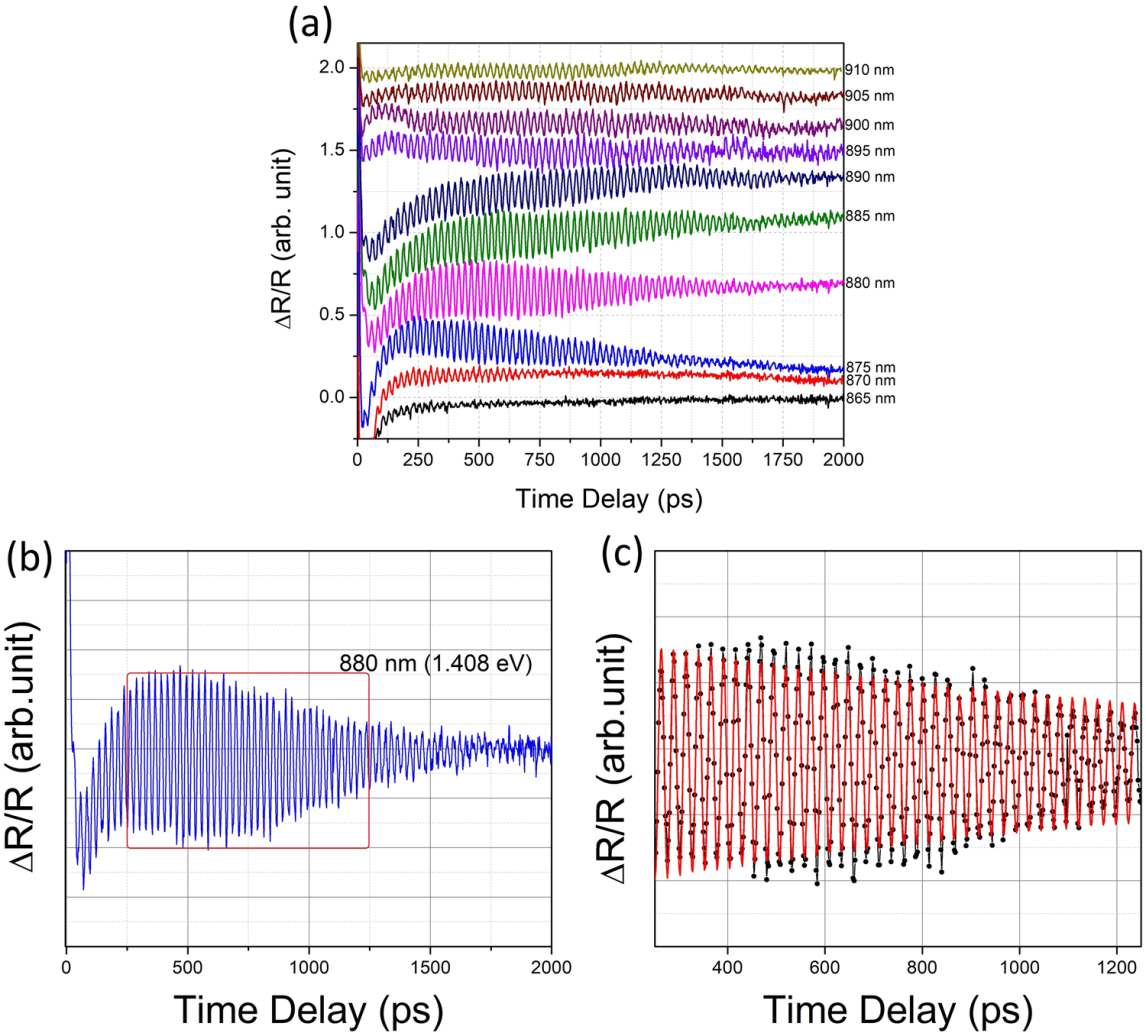
Extremely long-lived CLAP oscillations in intrinsic GaAs (100) single crystal wafers, which are clearly seen even after 2000 ps: (**a**) Observed using two-colour pump probe spectroscopy with 400 nm pump pulse and probe wavelengths close to the band gap from 865 to 910 nm, traces are presented with offset for clarity (**b**) enlarged representative CLAP oscillation observed at the probe wavelength of 880 nm is presented (**c**) enlarged representative oscillatory fit data to the equation, ${\rm{\Delta }}R/R=A\,sin(2\pi ft-\phi ){e}^{-t/{\tau }}$, *f* is the frequency of oscillation, *τ* is the decay constant and *φ* is the phase. The fit is obtained after subtracting the background arising from charge carrier relaxation, and neglecting the initial build-up of the CLAP oscillations and the noisy part at higher delay times. As may be seen from figure excellent fits were obtained for all probe wavelengths.

Optical pumping causes a sharp transient in the differential reflectance due to the photoexcitation process. Upon relaxation, after about 20 ps, this transient is followed by an oscillatory tail. Since the initial transient is not the subject of interest in the present work, we shall focus our attention on the oscillatory part following it. Figure 2(a) shows the oscillatory component of the measured *∆R/R* signal for different probe wavelengths. It can be seen that the CLAP oscillations begin to build-up typically after 50 ps. These phonon oscillations have large amplitude and are extremely long-lived, Fig. 2(b) for probe wavelengths close to the band gap (865–910 nm). These oscillations last for over 2000 ps and are by far the longest time for which they have been hitherto reported. After, subtracting the background arising from charge carrier relaxation, and neglecting the initial build-up of the CLAP oscillations and the noisy part at higher delay times, the oscillatory data was fitted to the equation,

2 $${\rm{\Delta }}R/R=A\,sin(2\pi ft-\phi ){e}^{-t/{\tau }}$$

In the above expression, *f* is the frequency of oscillation, *τ* is the decay constant and *φ* is the phase. As may be seen from the representative figure (Fig. 2(c)), Equation 2 provides an excellent fit obtained for all probe wavelengths. A summary of the fit parameters is presented in Table 2.

**Table 2:** Fit parameters of the experimental data of CLAP oscillations to the equation, ${\rm{\Delta }}R/R=Asin(2\pi ft-\phi ){e}^{-t/\tau }$.

Probe Wavelength (λ, nm)	Decay Constant (*τ*, ps)	Period (T, ps)	Phase shift (*φ*, rad.)	Max. Amplitude (a.u.)
865	915.11	25.008	2.295	0.055
870	987.62	25.394	1.218	0.177
875	1109.24	25.625	1.643	0.257
880	1034.58	25.854	0.923	0.252
885	1189.81	26.074	2.018	0.192
890	1712.84	26.292	0.175	0.123
895	1975.79	26.484	0.975	0.087
900	1787.67	26.685	0.482	0.056
905	1296.24	26.848	0.974	0.066
910	1511.45	27.056	0.867	0.012

**Figure 3:**
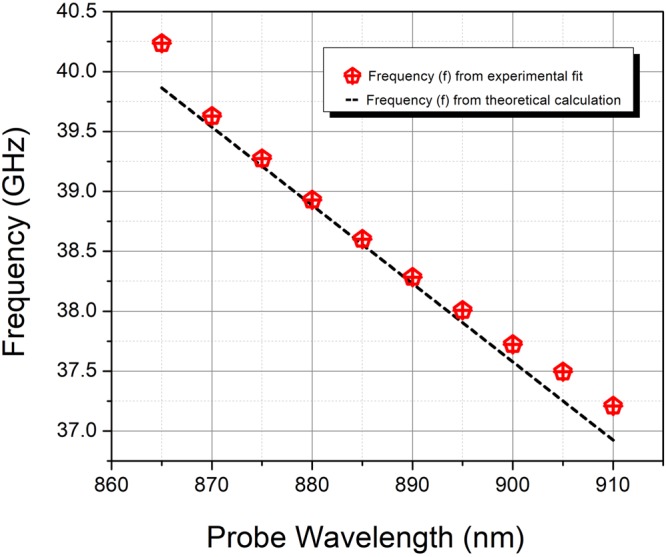
The variation of the frequency of CLAP oscillations obtained from the experimental fit with the linear fit obtained using the expression $f=\frac{1}{T}=\frac{2{V}_{s}n}{\lambda }$ where *λ* is the probe wavelength, *V*_s_ is velocity of sound and *n* is the refractive index, is presented for the probe wavelength ranges from 860 to 910 nm.

**Figure 4:**
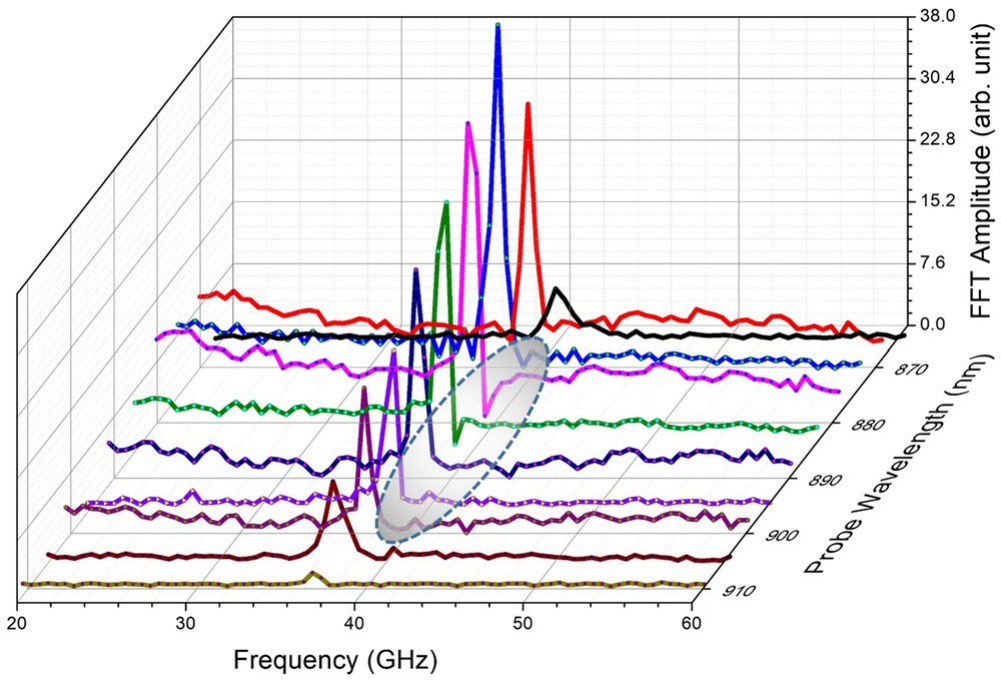
Fourier Transform (FT) of the time domain CLAP oscillations for the probe wavelengths close to the bandgap (865 to 910 nm) of bare GaAs (100) single crystal excited using the pump pulse at 400 nm. A strong deviation of spectra from symmetric Lorentzian lineshape to asymmetric Fano lineshape is observable for the oscillations obtained at probe wavelengths from 875 to 900 nm.

**Figure 5:**
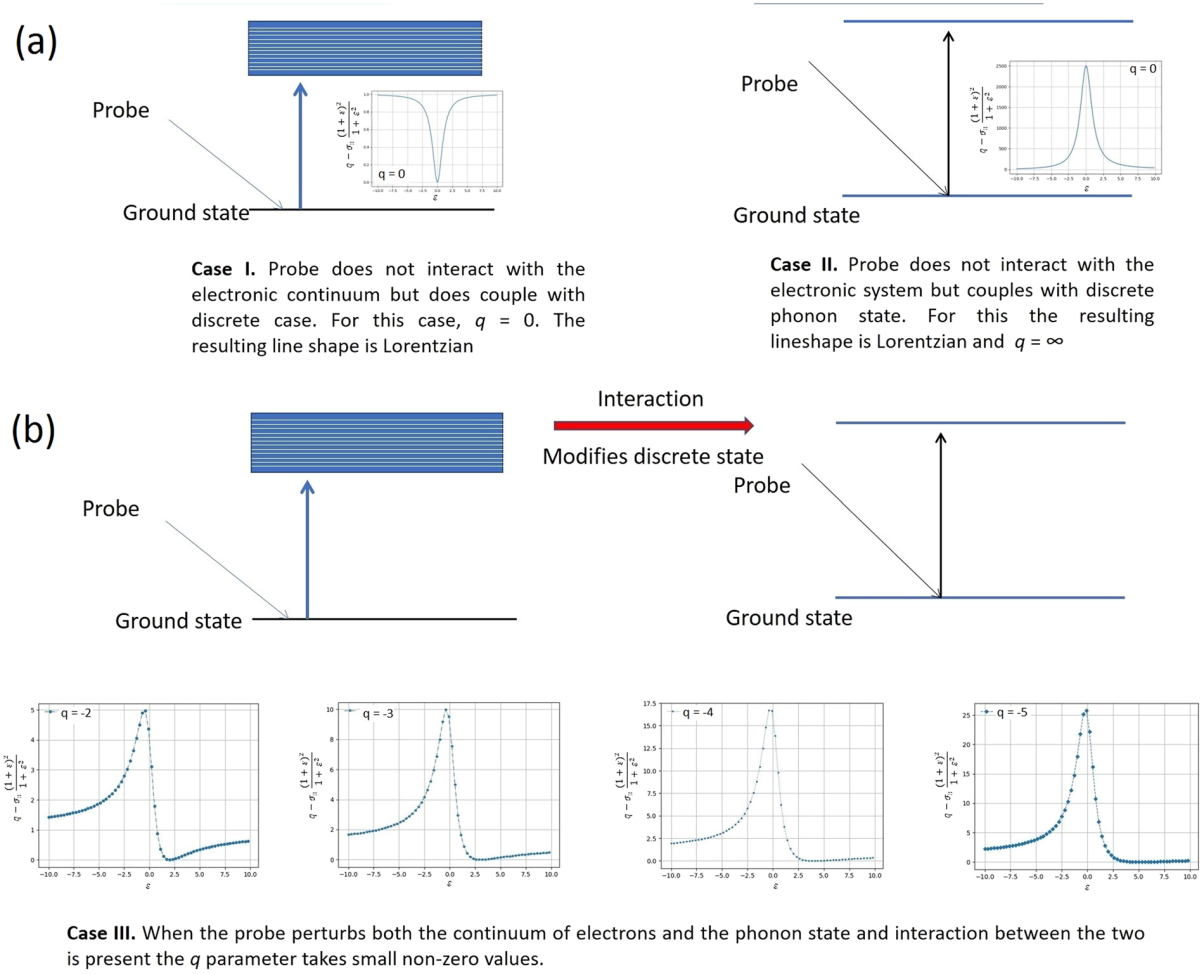
The probable mechanism of the interaction of probe beam with the continuum electronic states and discrete CLAP oscillation is presented (**a**) and the simulated Fano lineshape for some typical values of *q*, equivalent to the one observed from the experimental data are presented (**b**).

**Figure 6:**
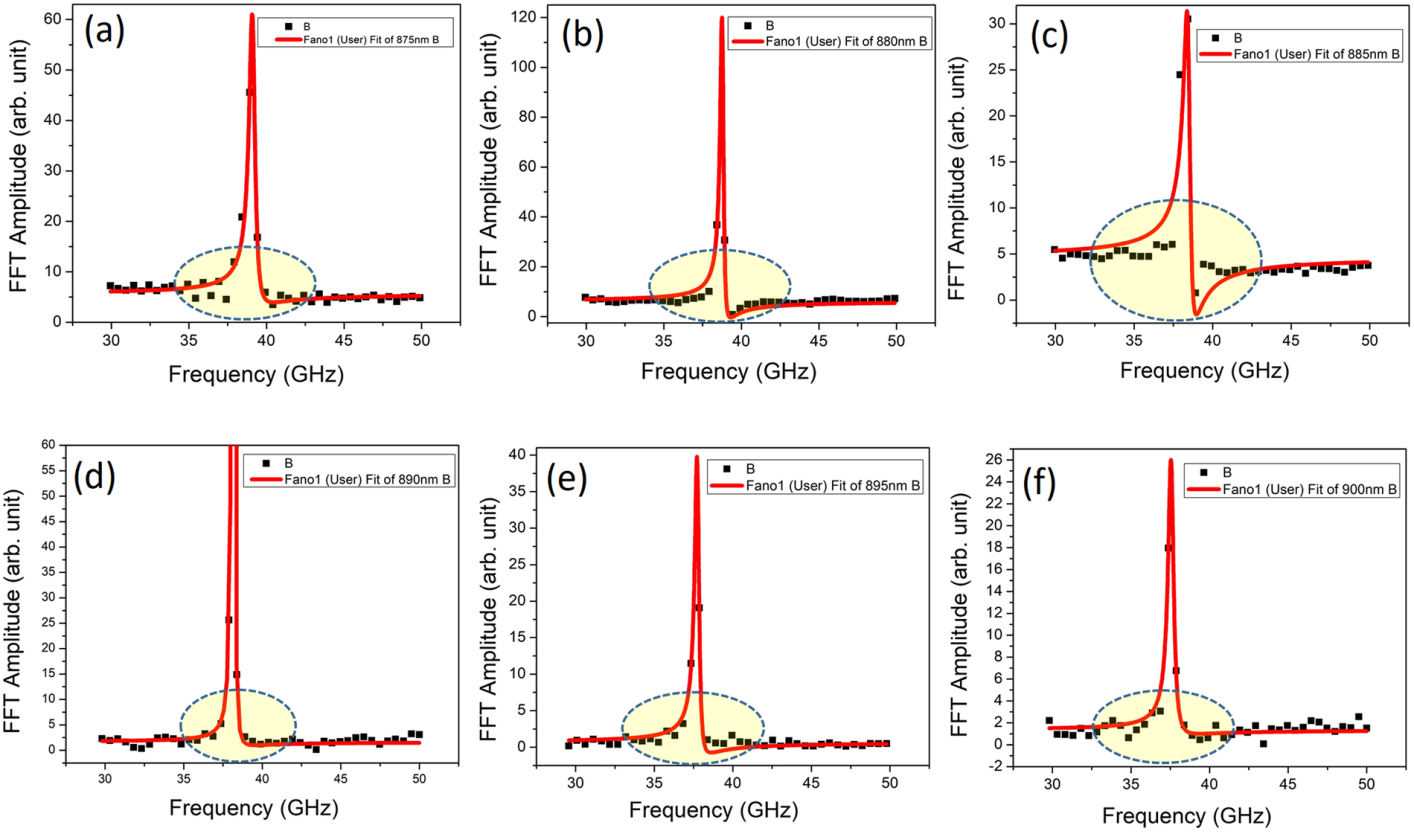
The fitted Fano lineshapes to the Fourier transform data obtained from the CLAP oscillations at different probe wavelengths (**a**) 875 (**b**) 880 (**c**) 885 (**d**) 890 (**e**) 895 and (**f**) 900 nm are presented.

**Figure 7:**
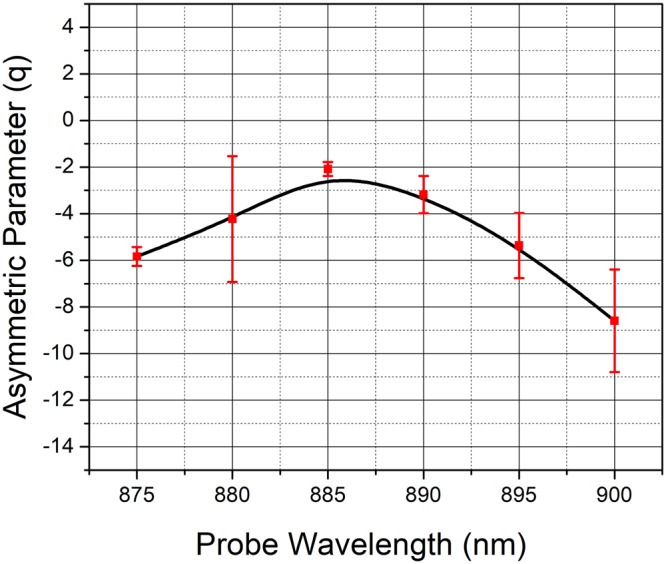
The asymmetry parameter obtained from the fit plotted as a function of probe wavelength is presented. The q value attains a maximum of −2 for the probe wavelength near the renormalized bandgap (885 nm) and on either side of the bandgap, the *q* value decreases.

The variation of the frequency of oscillation along with the linear fit using the expression $f=\frac{1}{T}=\frac{2{V}_{s}n}{\lambda }$ where *λ* is the probe wavelength, *V*_s_ is velocity of sound and *n* is the refractive index is shown in Fig. 3. For the fit, the value of *V*_*s*_ given in Table 1 and the known values of refractive index, *n* corresponding to different probe wavelength was used. From the figure, we see that the frequency of oscillation increases almost linearly from ~37 to 40 GHz. A good fit of the experimental data confirms that the periodic modulations of the probe pulse are caused by CLAP propagating in GaAs. From the Table 2, it is observable that the phase *φ* exhibits rapid variations with increase in probe wavelength. The rapid de-phasing with probe wavelength is suggestive of the interaction between the excited hot photo-carriers and coherent phonon oscillations which is not very evident in the time domain. The subtle features about the electron-phonon interaction can be better brought out from the lineshape analysis of the frequency domain data. Fourier transform (FT) of the time domain oscillations was thus carried out and is shown in Fig. 4. It may be observed that for the probe wavelengths close to the renormalized bandgap (~885 nm), there is a strong deviation of the spectral profile from the symmetric Lorentzian lineshape. In solids, the joint density of states is a quasi-continuum and the associated dipole response is effectively a superposition of delta functions over a range of frequencies. In the absence of any interaction, the exponential decay of the excited states results in a Lorentzian lineshape. The resonance cross section for the process is the typical symmetric Lorentzian/Breit-Wigner resonance^44^ given by the expression:

3 $$\sigma ({\epsilon })\propto \frac{1\,}{1+{{\epsilon }}^{2}}$$

where ${\epsilon }\,=(E-{E}_{r})/({\rm{\Gamma }}/2)\,$is energy, expressed in terms of the half-width of the line profile and *E*_*r*_, is the resonance energy. However, in the presence of an interaction between the quasi-continuous electronic states and the discrete phonon state, the cross section gets modified and is given by Fano expression of the form $\sigma ({\epsilon })=\frac{{(q+{\epsilon })}^{2}}{(1+{{\epsilon }}^{2})}$, where *q* is called the asymmetry parameter. Quantum mechanically, this interaction is well-described using Fano-Anderson Hamiltonian^45^ having the simple form:

4 $$H={E}_{0}{\mu }_{ph}^{\dagger }{\mu }_{ph}+{\sum }_{n}{E}_{n}^{el}{\nu }_{n}^{\dagger el}{\nu }_{n}^{el}+{\sum }_{n}{V}_{n}({\mu }_{ph}^{\dagger }{\nu }_{n}^{el}+{\nu }_{n}^{\dagger el}{\mu }_{ph})$$

here, the first and second terms on the right are associated with the discrete phonon and electronic states respectively. The last terms expresses the interaction between the two. The terms *E*_0_ and ${E}_{n}^{el}$ are excitation energies associated with phonon state and *n*^*th*^ electronic states respectively. The interaction matrix element between the discrete phonon states and those of the ${n}^{th}$ electronic state is represented by *V*_*n*_. As depicted in the schematic (Fig. 5a), the perturbing probe pulse interacts with the ground state of the system causing a transition with the associated matrix elements being expressed as ${M}_{\mu g}=\langle {\mu }_{ph}|\hat{M}|g\rangle$ and $\langle {v}_{n}^{el}|\hat{{M}}|g\rangle$ for the phonon and electronic states respectively. In the presence of such interaction, the intensity profile would have the Fano lineshape:

5 $$I(q,\omega )={I}_{B}(\omega )\frac{{(q+{\epsilon })}^{2}}{(1+{{\epsilon }}^{2})}\,$$

where the term ${I}_{B}(\omega )$ arises from the continuum background and the terms *q* and ${\epsilon }$ are the usual asymmetry parameter and normalized energy respectively. As limiting cases of the model, we have the scenario where the probe does not couple with the electronic system and the case where it does not couple with the discrete phonon system. Under the former condition the absolute value of the asymmetry parameter $q\to \infty \,\,$and under the latter condition $q\to 0$. Under either limit, a symmetric Lorentzian lineshape would be recovered as in the case for probe wavelength well below that associated with bandgap. We present the simulated Fano lineshapes for some typical values of *q* presented in Fig. 5(b), bringing out these features. For probe wavelengths close to the renormalized bandgap, we observe a pronounced asymmetry in the lineshape as clearly seen in Fig. 4. For emphasis; we have marked the relevant region with an elliptically shaded portion. The Fano lineshape fitted to the experimental data for various probe wavelengths are given in Fig. 6 for comparison. The asymmetry parameter obtained from the fit when plotted as a function of probe wavelength attains a maximum of −2 for the probe wavelength close to the bandgap energy as shown in Fig. 7. On either side of the bandgap, the *q* value decreases and the corresponding lineshape becomes nearly a Lorentzian as expected from the theory. The lineshape for 905 nm probe wavelength is nearly symmetric as would be expected for larger values of *q*, indicating that the probe has little interaction with the electronic states. In the presence of a weak coupling however, the absolute value of the *q*-parameter would take on small values. This is indeed the case for probe wavelengths between 875–890 nm where Fano resonance is observed. Herein, we may note that the sign of the *q* parameter is negative, which implies that the polarizability of the CLAP and continuum states is of the opposite sign. Further, since the minimum in the line shape occurs on the side higher than the resonant energy close to the band gap, we infer that the phase shift between the continuum and discrete mode is between $\frac{3n\pi }{4}$ and *nπ* as the line shape becomes symmetric at *nπ* and $(n+\frac{1}{2})\pi$. Thus, from the nature of the line shape, we see that the CLAP oscillations and electronic transitions strongly depend on the wavelength of the probe in terms of the value of $q\,\,$and bearing a definite shift in the phase.

## Conclusion

In summary, long lived coherent acoustic phonon oscillations in pure GaAs were observed in two colour pump-probe spectroscopy. The lineshape of the oscillations are found to have a pronounced asymmetry close to the bandgap. The asymmetry is inferred to occur due to Fano resonance between the continuum of electronic transition and the CLAP state. It is interesting to note that the line-shape is asymmetric for probe wavelengths between 875–895 nm and becomes symmetric only after 900 nm. We wish to stress that clear evidence of Fano resonance with respect to phonon oscillations has been presented in very few cases, most notably in Bi and Zn. Though, asymmetry of Raman spectral lineshape has been reported for the case of optical phonons for GaAs, the reason why Fano-resonance has not been reported earlier for CLAP is two-fold: 1. Most of the reported CLAP oscillation studies in GaAs investigations have not been carried out on pristine undoped GaAs but have been done with doped specimens, specimens with a capping layer or on specimens bonded to a substrate to induce strain. 2. The data is presented in the time domain more often and the frequency domain not analyzed. Further in the present work, optical pumping is done with photon energies way above the band gap energy and with high fluence, thereby creating a larger contribution to the overall stress.

## Data Availability

We certify that all data collected and presented in the manuscript will be made available unconditionally as and when required.
